# Iron (FeII) Chelation, Ferric Reducing Antioxidant Power, and Immune Modulating Potential of *Arisaema jacquemontii* (Himalayan Cobra Lily)

**DOI:** 10.1155/2014/179865

**Published:** 2014-05-06

**Authors:** Rasleen Sudan, Madhulika Bhagat, Sahil Gupta, Jasvinder Singh, Anupurna Koul

**Affiliations:** ^1^School of Biotechnology, University of Jammu, Jammu, Jammu and Kashmir 180006, India; ^2^Department of Pharmacology, Indian Institute of Integrative Medicine, Jammu, Jammu and Kashmir 180001, India

## Abstract

This study explored the antioxidant and immunomodulatory potential of ethnomedicinally valuable species*, namely, Arisaema jacquemontii *of north-western Himalayan region. The tubers, leaves, and fruits of this plant were subjected to extraction using different solvents. *In vitro* antioxidant studies were performed in terms of chelation power on ferrous ions and FRAP assay. The crude methanol extract of leaves was found to harbour better chelating capacity (58% at 100 **μ**g/mL) and reducing power (FRAP value 1085.4 ± 0.11 **μ**MFe^3+^/g dry wt.) than all the other extracts. The crude methanol extract was thus further partitioned with solvents to yield five fractions. Antioxidant study of fractions suggested that the methanol fraction possessed significant chelation capacity (49.7% at 100 **μ**g/mL) and reducing power with FRAP value of 1435.4 **μ**M/g dry wt. The fractions were also studied for immune modulating potential where it was observed that hexane fraction had significant suppressive effect on mitogen induced T-cell and B-cell proliferation and remarkable stimulating effect on humoral response by 141% and on DTH response by 168% in immune suppressed mice as compared to the controls. Therefore, it can be concluded that *A. jacquemontii* leaves hold considerable antioxidant and immunomodulating potential and they can be explored further for the identification of their chemical composition for a better understanding of their biological activities.

## 1. Introduction

Natural products from plants have gathered significant attention due to the recent evidences suggesting their capacity to ameliorate oxidative stress and other related diseases. Plant secondary metabolites such as phenolics, flavonoids, and terpenoids play an important role as natural antioxidants and immunomodulators [1]. In biological systems, it is often considered that reactive oxygen species (ROS) originate from the interaction of iron with enzymatically and/or nonenzymatically generated superoxide (O_2_^∙−^, Haber-Weiss reaction) and/or hydrogen peroxide (H_2_O_2_, Fenton reaction) [2]. The human body harbours enzymatic defence mechanisms to counter oxidative stress induced by free radicals which involve superoxide dismutase, catalase, and glutathione peroxidase. Apart from innate defence system, synthetic drugs are also available which improve the capacity of the body to counter oxidative stress and other diseases. But owing to the harmful side effects of synthetic drugs, research on natural products has taken a leap in recent years.

Phytochemicals have also proved to enhance immunity by modulating the innate and adaptive immune responses. Immunomodulation using medicinal plants can provide an alternative to conventional chemotherapy for a variety of diseases, especially when the host defence mechanism has to be activated under conditions of impaired immune response or when a selective immunosuppression is desired in situations such as autoimmune disorders. Several types of immunomodulators have been identified, including substances isolated and purified from natural sources such as plants including microorganisms. Polyphenolic compounds such as phenolic acids, flavonoids, anthocyanidins, and tannins, produced as secondary metabolites by plants, possess remarkable antioxidant and immunomodulatory activities [3].

The genus* Arisaema* (commonly known as “Cobra Lilies”) is made up of more than 250 herbaceous species, which are distributed throughout temperate to tropical areas.* Arisaema sp.* are traditionally documented and are known to be used by the indigenous people of India and China as antinematodal, anti-inflammatory, analgesic, and antidote and also for treating rheumatoid arthritis [4–7].* A. heterophyllum*,* A. peninsulae*,* A. robustum*,* A. consanguineum*,and* A. japonicum *were frequently used in Chinese herbal medicine as an anticonvulsant [8]. Rhizome of* A. jacquemontii* grounded with edible oil forms a paste which is used for massage to regain muscular strength and in skin problems such as blisters and pimples [9].

*Arisaema jacquemontii* is commonly found in the Himalayan forests at an altitude of 2,300–4,300 m. It also occurs in the Nilgiri Hills in southern India and the Khasi Hills region of north-east India. This plant has not been scientifically evaluated for its antioxidant and immunomodulatory potential. Owing to the lack of availability of substantial reports, the present study was planned to explore the biological potential of this traditionally documented plant.

## 2. Materials and Methods

### 2.1. Preparation of Extracts and Fractions

*A. jacquemontii* was collected from high altitude (2500–3500 m) from north-western Himalayan region and was identified by the taxonomist of University of Jammu. The leaves, fruits, and tubers were dried separately and grounded in a blender to make fine powder. A total of 100 g powder of each of the leaves, fruits, and tubers were extracted with 500 mL of three different solvents, that is, methanol (AJL1), water (AJL2), and chloroform (AJL3). Methanol and chloroform extracts were obtained by continuous stirring at room temperature for 6 h whereas water extract was prepared at 60°C overnight. This treatment was repeated thrice and the extracts were pooled, filtered, and evaporated using rotary vacuum evaporator. Active crude extract was further sequentially partitioned with different solvents like hexane (AJL1-H), chloroform (AJL1-C), ethyl acetate (AJL1-E), acetone (AJL1-A), and methanol (AJL1-M) to obtain fractions (Figure 3). All the fractions were freeze-dried using rotary vacuum evaporator and lyophilized.

### 2.2. Antioxidant Studies

#### 2.2.1. Chelation Power on Ferrous (Fe^2+^) Ions

The chelating effect on ferrous ions of the prepared extracts was estimated by the method of Dinis with slight modifications [10]. Briefly, 100 *μ*L of each test sample (1 mg/mL) was taken and raised to 3 mL with methanol. 740 *μ*L of methanol was added to 20 *μ*L of 2 mM FeCl_2_. The reaction was initiated by the addition of 40 *μ*L of 5 mM ferrozine into the mixture, which was then left at room temperature for 10 min and then the absorbance of the mixture was determined at 562 nm.

*(i) Ferric Ion Reducing Antioxidant Power (FRAP Assay). *FRAP activity was measured according to the method of Benzie and Strain [11]. Briefly, acetate buffer (300 mM, pH 3.6), TPTZ (2,4,6-tripyridyl-s-triazine) 10 mM in 40 mM HCl and FeCl_3_·6H_2_O (20 mM) were mixed in the ratio of 10 : 1 : 1 to obtain the working FRAP reagent. Test sample (0.5 mL) was mixed with 3 mL of working FRAP reagent and absorbance was measured at 593 nm after vortexing. Methanol solutions of FeSO_4_·7H_2_O ranging from 100 to 2000 *μ*M were prepared and used for the preparation of the calibration curve of known Fe^2+^ concentration. The parameter equivalent concentration was defined as the concentration of antioxidant having a Ferric-TPTZ reducing ability equivalent to that of 1 mM FeSO_4_·7H_2_O.

### 2.3. Acute Toxicity Study

Acute toxicity study of fractions was conducted as per OECD guidelines 420 (fixed dose procedure) using Swiss mice. Each animal was administered test samples at a dose of 2000 mg/kg by oral route. The animals were observed for any changes continuously for the first 4 h and up to 24 h for any mortality. The animals were then kept for 14 days to observe daily cage side observations and mortality [12].

### 2.4. Immunomodulatory Studies

#### 2.4.1. Mitogen Activity Test by MTT Assay

Mitogen activity test was based on the method described by Mosmann [13]. Animals (balb/c mice, 18–22 g) were sacrificed; their spleens were removed in sterile conditions. A single cell suspension was prepared in 5 mL of incomplete RPMI. The cell suspension was centrifuged at 1200 rpm for 10 min and supernatant was discarded. RBCs were lysed by Trisammonium chloride treatment. The cells were centrifuged at 1200 rpm for 10 min, after centrifuging, supernatant was discarded and cell pellet was resuspended in complete RPMI. The viability of cells was checked with trypan blue. 1 × 10^6^ cells/mL suspension was prepared and 100 mL of it was poured in each well of 96-well microtitre plate. An aliquot of 50 mL of standard mitogens, that is, Concanavalin A (Con A) (1/4) 10 mg/mL and Lipopolysaccharide (LPS) (1/4) 10 mg/mL, and test materials were added according to the experimental setup. The extracts in different concentrations (10^−4^, 10^−5^, and 10^−6^ M) were dissolved in DMSO and added to each well of flat bottom microtitre 96-well plate. Plates were placed on a shaker for 5 min. The plates were incubated for 48 h in CO_2_ incubator (37°C, 5% CO^2^, and 90% relative humidity). After 48 h of incubation, plates were taken out from the CO_2_ incubator and reading was taken on ELISA plate reader at 540 nm. Thereafter, 10 *μ*L of MTT solution (5 mg/mL in PBS) was added to each well. The contents were placed on a shaker for 5 min and plates were incubated for 4–6 h in CO_2_ incubator (37°C, 5% CO^2^, and 90% relative humidity) to allow the MTT to be metabolized. After incubation, the plates were inverted on a paper towel to remove the medium. The formazan crystals (MTT byproduct) were resuspended in 100 mL DMSO and reading was measured at a wavelength of 570 nm.

#### 2.4.2. Effect on Humoral and Cellular Response in Immune Suppressed Mice

Swiss albino mice (*Mus musculus*) 10–12 weeks old, with 20–25 g body weight, and male Charles Foster rats (*Rattus norvegicus*) 10–12 weeks, with 100–150 g body weight, in groups of six were employed for study. In every experiment, one group of animals was used as a vehicle control while another received a standard drug Azathioprine (Aza). The test sample was freshly prepared as a homogenised suspension in 1% w/v acacia gum administered orally daily once a day for the duration of the experiment. 

*(1) Antigen (SRBC)*. Fresh sheep red blood cells (SRBC) collected aseptically from jugular vein of sheep were stored in cold sterile Alsever's solution, washed three times with pyrogen free sterile normal saline (0.9% NaCl w/v), and adjusted to a concentration of 5 × 10^9^ cells/mL for immunization and challenge at the required time schedule.

*(2) Humoral Antibody Response (Hab). *Groups of six mice each were immunized by injecting 0.2 mL of 5 × 10^9^ SRBC/mL intraperitoneally (i.p.) on day 0 and challenged 7 days later by injecting an equal volume of SRBC i.p. Blood samples were collected on day +7 (before challenge) for primary antibody titre. Hemagglutination antibody titres were determined following the microtitration technique described by Nelson and Mildenhall [14]. The value of the highest serum dilution causing haemagglutination was taken as a titre. BSA saline alone served as a control.

*(3) Delayed Type Hypersensitivity Response (DTH). *The method of Doherty was followed to assess SRBC induced DTH response in mice [15]. Mice were immunized by injecting 20 *μ*L of 5 × 10^9^ SRBC/mL subcutaneously into the right hind foot pad. Seven days later, the thickness of the left hind foot was measured with a spheromicrometer (0.01 mm pitch) and was considered as a control. These mice were then challenged by injecting the same amount of SRBC intradermally into the left hind foot pad. The foot thickness was measured again at 0, 4, and 24 hr after challenge.

### 2.5. Phytochemical Analysis

The fractions obtained from the crude methanol extract of leaves were also investigated for the presence of secondary metabolites like terpenoids, coumarins, glycosides, quinones, saponins, tannins, anthraquinones, alkaloids, phenols, and flavonoids.

### 2.6. Statistical Analysis

All experiments were carried out in triplicate. Data values are expressed as mean ± standard deviation.

## 3. Results and Discussion

Diverse species of* Arisaema* hold ethnomedicinal importance in different areas of Asian subcontinent for treating various medical ailments. The rhizomes or tubers of* A. calcareum*,* A. serratum*,* A. asperatum*,* A. heterophyllum,* and* A. amurense *are used as analgesic, antitumor, and pesticide agents in traditional Chinese medicine [16]. Chinese herbal traditional medicine system uses* A. cumbile* for treating dementia and neurological symptoms [17]. Later on, studies explained that* A. cumbile* inhibits the production of proinflammatory cytokines including interleukin (IL-)1**β**, IL-6, and tumor necrosis factor (TNF-)**α** [18]. Antihepatotoxic cerebrosides have also been isolated from* A. amurense* [8].* A*.* erubescens* is a widely distributed species in China and it is used as a medicinal herb against damp phlegm, convulsions, and swelling [19]. Pharmacological study has also proved that this species harbors anticonvulsant and anticancer effects [20]. Paeonol, a phenolic compound which possesses antimutagenic, anticonvulsant, and anti-inflammatory activities, has been isolated from* A. erubescens* [21].

However, sufficient scientific data regarding the biological potential of* A. jacquemontii* is still lacking. In view of this fact, our study focussed on unearthing the antioxidant and immunomodulating potential of* A. jacquemontii, *which is found in abundance in the high altitude forests of Himalayas. Extracts of each of tubers, leaves, and fruits of* A. jacquemontii* were prepared in chloroform, methanol, and water and tested for antioxidant potential by undertaking* in vitro* chemical assays, mainly chelation power on ferrous ions and ferric ion reducing antioxidant power (FRAP).

**Figure 1:**
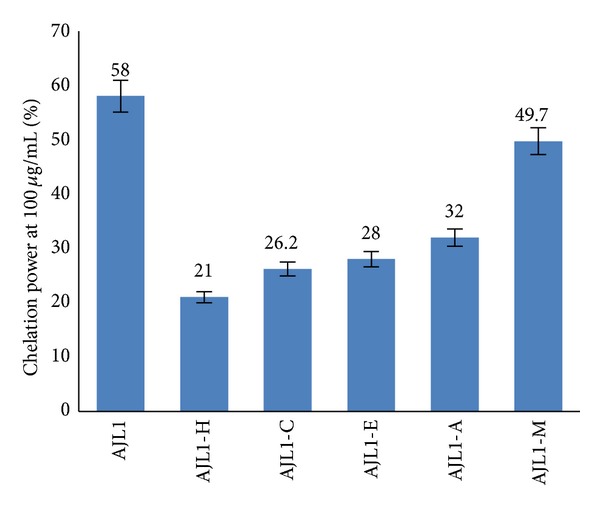
Chelation power on ferrous ions of* A. jacquemontii* crude methanol leaf extract and its fractions. AJL1: crude methanol leaf extract, AJL1-H: hexane fraction, AJL1-C: chloroform fraction, AJL1-E: ethyl acetate fraction, AJL1-A: acetone fraction, and AJL1-M: methanol fraction.

Most reactive oxygen species (ROS) are generated as by-products during mitochondrial electron transport and other metabolic reactions. In addition, ROS are formed as necessary intermediates of metal catalyzed oxidation reactions. The transition metal ion Fe^2+^ possesses the ability to perpetuate the formation of free radicals by gain or loss of electrons. Therefore, the reduction of the formation of reactive oxygen species can be achieved by the chelation of metal ions with chelating agents. Chelation power assay was carried out to assess the chelation capacity of the crude extracts which illustrated that the crude methanol extract of leaves of* A. jacquemontii* possessed remarkable chelation power at 100 *μ*g/mL (58%) as compared to tuber (12%) and fruit extracts (34%) (Table 1). However, the aqueous and chloroform extracts of tubers, leaves, and fruits showed negligible activity. Therefore, active crude methanol extract of leaves was subjected to sequential fractionation yielding five fractions, that is, hexane (AJL1-H), chloroform (AJL1-C), ethyl acetate (AJL1-E), acetone (AJL1-A), and methanol (AJL1-M). Chelation power of fractions was also analysed and it was observed that the methanol fraction exhibited significant capacity to chelate ferrous ions in comparison to other fractions with the value of 49.7% at 100 *μ*g/mL (Figure 1). Excess of metal ions can lead to various anomalies in the body. The iron (II) chelating activity of plant extracts is of great significance, because it has been proposed that the transition metal ions contribute to the oxidative damage in neurodegenerative disorders like Alzheimer's and Parkinson's diseases [22]. Also, chelation therapy is a common practice of neutralising iron overload in the body especially in cases of treatment of Thalassemia and other anemias [23]. The current scenario suggests that the chelation therapy makes use of synthetic compounds which have certain side effects as well. Therefore, chelation of metal ions by natural phytochemicals from* Arisaema sp.* can prove to be of therapeutic importance.

**Figure 2:**
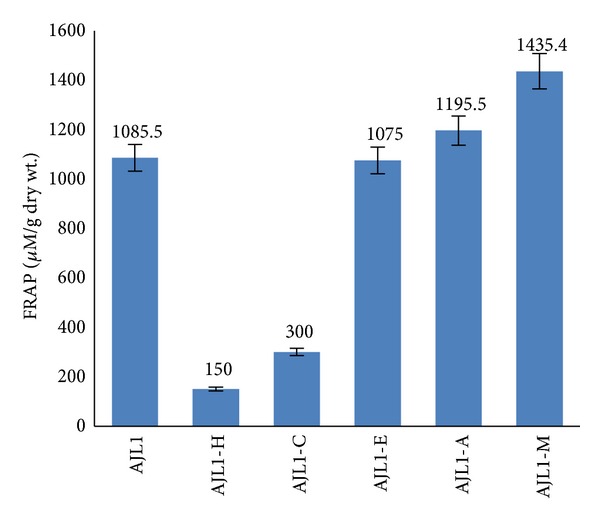
Ferric reducing antioxidant power (FRAP) of* A. jacquemontii* crude methanol leaf extract and its fractions. AJL1: crude methanol leaf extract, AJL1-H: hexane fraction, AJL1-C: chloroform fraction, AJL1-E: ethyl acetate fraction, AJL1-A: acetone fraction, and AJL1-M: methanol fraction.

**Figure 3:**
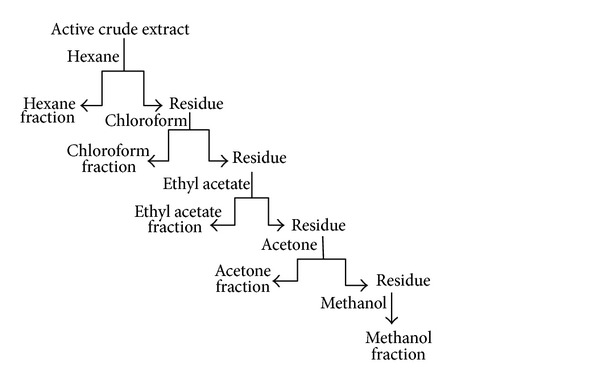
Flowchart of sequential partitioning of active crude methanol extract of leaves of* A. jacquemontii* to obtain fractions.

Another assay, that is, ferric reducing antioxidant power (FRAP), was conducted on all the extracts and fractions of* A. jacquemontii *to confirm its antioxidant potential. In this assay, reduction of ferric tripyridyl triazine (Fe^3+^-TPTZ) complex to ferrous form which has an intense blue colour can be monitored by measuring the change in absorption at 593 nm. The results of this experiment were similar to that of chelation power assay; that is, crude methanol extract of* A. jacquemontii *leaves showed noteworthy FRAP activity with reducing the value of 1085.4 ± 0.11 *μ*M/g dry wt. However, tuber and fruit methanol extracts possessed very low FRAP activity with values of 37 ± 0.021 *μ*M/g dry wt. and 635.4 ± 0.032 *μ*M/g dry wt., respectively (Table 1). Also, further FRAP analysis of fractions showed that methanol fraction (AJL1-M) possessed better reducing power with FRAP value of 1435.4 *μ*M/g dry wt. in comparison to other fractions (Figure 2).

Antioxidant studies suggested that the crude methanol extract of leaves (AJL1) and its subsequent methanol fraction (AJL1-M) possessed promising chelating and reducing antioxidant power as compared to all the other extracts of tubers and fruits. All the fractions were also studied for the effects of acute toxicity and behavioural changes using Swiss mice as per OECD guidelines 420 (Fixed dose procedure). It was observed that there was no mortality and noticeable behavioural changes in treated animals as compared to control animals. All the fractions were found to be safe up to 2000 mg/kg body weight p.o.

Many medicinal plants are a rich source of substances which induce para-immunity, non-specific activation of granulocytes, macrophages, natural killer cells and the complement system. Confirming the safety levels of all the fractions by acute toxicity study in mice, they were further analysed for immune modulating potential by studying the effect on Con A and LPS induced murine lymphocyte proliferation (MTT assay). Concanavalin A and Lipopolysaccharide (LPS) are the mitogens which activate T-cell and B-cell proliferation, respectively, in the splenocytes. MTT [3-(4,5-dimethylthiazol-2-yl)-2,5-diphenyltetrazolium bromide] assay is based on the ability of a mitochondrial dehydrogenase enzyme from viable cells to cleave the tetrazolium rings of the pale yellow MTT and form a dark blue formazan crystals which is largely impermeable to cell membranes, thus resulting in its accumulation within healthy cells. The number of surviving cells is directly proportional to the level of the formazan product created which can then be quantified using a simple colorimetric assay [13]. Hexane (AJL1-H) and ethyl acetate fraction (AJL1-E) showed significant suppressive effect on LPS induced B-cell proliferation and moderate effect on Con-A induced T-cell proliferation. However, methanol fraction (AJL1-M) also depicted considerable immune suppression on both B-cell and T-cell proliferation (Table 2). Immune suppression finds application in the treatment of autoimmune disorders such as rheumatoid arthritis and multiple sclerosis [24].

Furthermore, the effect of fractions on humoral and cell mediated immune response in immune suppressed mice was also studied (Table 3). The results after 7 days of oral administration of fractions to the immune suppressed mice showed some interesting results. The hexane fraction (AJL1-H) was observed to enhance the humoral and cell-mediated immune response in immune suppressed mice (Table 3). The humoral antibody titre at the concentration of 100 mg/mL was observed to be 141% which was more than that of standard drug, Levamisole (133%). Also, AJL1-H showed noteworthy positive DTH response at all three concentrations of 100 mg/mL (168%), 50 mg/mL (100%), and 25 mg/mL (128%). Also, acetone fraction (AJL1-A) showed moderate humoral and DTH stimulating responses at concentration of 100 mg/mL and 50 mg/mL and methanol fraction (AJL1-M) demonstrated immune stimulating activity only at concentration of 25 mg/mL (Table 3). The other fractions were not found to be much effective as immune stimulants. Therefore, it can be deduced from the study that hexane fraction of* A. jacquemontii *leaves (AJL1-H) possesses significant immune stimulating potential as it showed potent abrogative effect on humoral antibody response and delayed type hypersensitivity response in immune suppressed balb/c mice and these observations are suggestive of possible therapeutic usefulness in immune compromised patients.

The phytochemical analysis revealed that all the fractions contain high amount of terpenoids. Coumarins, quinones, and glycosides were present in moderate amount (Table 4). Alkaloids, anthraquinones, and flavonoids were found in low quantity and phenols were detected in moderate amount only in acetone and methanol fractions. This was a preliminary analysis of the phytoconstituents present in the fractions. However, the phytochemicals responsible for the antioxidant and immune modulating potential of* A. jacquemontii* leaves still need to be identified.

## 4. Conclusion

This work is an attempt to identify the biological potential of* A. jacquemontii *growing in the Himalayan region for the first time. The methanol fraction obtained from crude methanol extract of leaves was observed to harbour considerable antioxidant potential and, in case of immune modulating studies, the hexane fraction was observed to show remarkable immune suppressive as well as immune stimulating potential, which could prove to be of immense value in autoimmune diseases as well as in immune compromised patients. However, the phytoconstituents responsible for their significant activity are still unknown. The results obtained in this work provide the basis for designing future experimentation on this species for better understanding of its antioxidative and immunomodulatory system and discovering the phytochemicals responsible for these properties.

## Figures and Tables

**Table 1:** Chelation power on ferrous ions and ferric reducing antioxidant power (FRAP) of *A. jacquemontii* crude extracts.

Extracts	Chelation power on ferrous ions (% at 100 µg/mL)			FRAP (*μ*M/g dry wt.)		
*A. jacquemontii *	Tuber	Leaves	Fruit	Tuber	Leaves	Fruit
Methanol (AJL1)	12	58	34	37 ± 0.021	1085.4 ± 0.11	635.4 ± 0.032
Aqueous (AJL2)	9	28	20	77.4 ± 0.41	805 ± 0.26	159.5 ± 0.015
Chloroform (AJL3)	13	32	22	na	na	na

*Values are expressed as mean ± standard deviation. na: not active.

**Table 2:** Effect of different concentrations of fractions of crude methanol extract of *A. jacquemontii* leaves on Con A and LPS induced murine lymphocyte proliferation.

Serial number	Samples	Conc. (M)	Con A mean ± S.E.	Con A induced T-cell proliferation rate (%)	LPS mean ± S.E.	LPS induced B-cell proliferation rate (%)
(1)	Control	—	0.75 ± 0.50	—	1.92 ± 0.05	—

(2)	**AJL1-H**	10^−4^	0.34 ± 0.04	−54.66	0.39 ± 0.01	**−79.68**
		10^−5^	0.76 ± 0.03	+1.33	0.44 ± 0.01	**−77.08**
		10^−6^	0.58 ± 0.02	−22.66	0.60 ± 0.01	**−68.75**

(3)	AJL1-C	10^−4^	0.69 ± 0.12	−22.47	0.82 ± 0.17	−16.32
		10^−5^	0.81 ± 0.08	−8.98	0.85 ± 0.06	−13.26
		10^−6^	0.82 ± 0.14	−7.86	0.83 ± 0.09	−15.30

(4)	**AJL1-E**	10^−4^	0.60 ± 0.11	−20.00	0.36 ± 0.01	**−81.25**
		10^−5^	0.54 ± 0.03	−28.00	0.48 ± 0.01	**−75.00**
		10^−6^	0.71 ± 0.00	−5.33	0.59 ± 0.01	**−69.27**

(5)	AJL1-A	10^−4^	0.43 ± 0.02	−42.66	0.54 ± 0.30	−72.21
		10^−5^	0.71 ± 0.05	−5.33	0.62 ± 0.30	−67.75
		10^−6^	0.64 ± 0.03	−14.66	0.60 ± 0.17	−68.12

(6)	**AJL1-M**	10^−4^	0.59 ± 0.14	**−33.70**	0.48 ± 0.05	**−75.00**
		10^−5^	0.70 ± 0.01	**−21.34**	0.52 ± 0.04	**−72.91**
		10^−6^	0.59 ± 0.14	**−33.70**	0.55 ± 0.09	**−71.35**

+ indicates immune stimulant agents, while − indicates immunosuppressive agents. Results are mean standard error (SE) of three separate experiments. The bold values are shown for those compounds which have proved to be active and those in normal font represent the least significant.

**Table 3:** Effect of active methanol fraction of *A. jacquemontii *leaves on humoral and cell mediated immune response.

Sample	Conc. mg/kg p.o.	Antibody titre Mean ± S.E.	% Activity	DTH Mean ± S.E.	% Activity
Control	—	6.5 ± 0.21	—	0.80 ± 0.16	—

Cyclophosphamide	200	4.5 ± 0.21	−30.7	—	—

Cyclosporine	5	—	—	0.35 ± 0.10	−56.25

Levamisole	2.5	7.16 ± 0.22	+133	1.11 ± 0.22	+168

**AJL1-H (mg/mL)**	**100**	7.33 ± 0.21	**+141**	1.11 ± 0.22	**+168**
	**50**	5.83 ± 0.16	**+67**	0.80 ± 0.16	**+100**
	**25**	3.83 ± 0.16	**+33**	0.91 ± 0.16	**+124**

AJL1-C (mg/mL)	100	5.83 ± 0.16	+67	0.48 ± 0.21	−29
	50	4.66 ± 0.21	+8	0.45 ± 0.22	+23
	25	5.16 ± 0.16	+33	0.60 ± 0.20	+56

AJL1-E (mg/mL)	100	5.66 ± 0.21	+58	0.63 ± 0.21	+63
	50	5.16 ± 0.16	+33	0.41 ± 0.16	+14
	25	5.33 ± 0.21	+42	0.50 ± 0.22	+34

AJL1-A (mg/mL)	100	6.33 ± 0.21	+91	0.80 ± 0.16	+100
	50	6.16 ± 0.40	+83	0.83 ± 0.16	+106
	25	5.33 ± 0.21	+42	0.53 ± 0.21	+40

AJL1-M (mg/mL)	100	5.16 ± 0.16	+33	0.58 ± 0.16	+52
	50	4.16 ± 0.16	−17	0.38 ± 0.22	−7
	25	6.5 ± 0.22	+100	0.93 ± 0.21	+128

Values are expressed as mean ± standard deviation.

**Table 4:** Preliminary phytochemical analysis of fractions of crude methanol extract of *A. jacquemontii* leaves.

Phytochemicals	Fractions				
	Hexane (AJL1-H)	Chloroform (AJL1-C)	Ethyl acetate (AJL1-E)	Acetone (AJL1-A)	Methanol (AJL1-M)
Terpenoids	++++	++	+++	+++	+++
Coumarins	+	++	+	+	+
Quinones	++	++	+	+	+
Saponins	−	−	−	−	−
Glycosides	++	+	+++	++	−
Tannins	−	−	−	−	−
Alkaloids	+	+	−	+	+
Anthraquinones	+	+	−	−	−
Phenols	−	−	+	++	++
Flavonoids	−	−	−	−	+

++++ indicates high amount; ++ indicates moderate amount; + indicates low amount; − indicates absence.
